# The complete Chloroplast Genome of *Dianthus Helenae*, an Endemic Species with Medicinal Potential from the Nuratau Mountains, Uzbekistan

**DOI:** 10.12688/f1000research.180038.4

**Published:** 2026-08-21

**Authors:** Ibrokhimjon Ergashov, Farkhodjon Mingboev, Farruhbek Rasulov, Diyorjon Hamrayev, Davronjon Sultonov, Risolat Achilova, Saidkamol Xaydarov, Bakhrom Khujamkulov, Feruza Utayeva, Oysha Jabborova, Ziyoviddin Yusupov

**Affiliations:** 1 Institute of Botany, Academy of Sciences of Uzbekistan, Tashkent, Uzbekistan; 2 Andijan State Medical Institute, Andijan State Medical Institute, Andijan, Uzbekistan; 3 Department of Botany and Biotechnology, Fergana State University, Fergana, Uzbekistan; 4 Department of Foreign Language Teaching Methodology, Bukhara State Pedagogical Institute, Bukhara, Uzbekistan; 5 Karshi State University, Kashkadarya, Uzbekistan; 6 Bukhara State University, Bukhara, Uzbekistan; 7 Bukhara State Medical Institute named after Abu Ali ibn Sino, Bukhara, Uzbekistan

**Keywords:** Caryophyllaceae, chloroplast genome, endemic species, Dianthus

## Abstract

*Dianthus helenae* Vved. is an endemic medicinal species of the Nuratau Mountains, Uzbekistan, and its genomic resources have remained largely unavailable. In this study, we sequenced, assembled, and characterized the complete chloroplast genome of *D. helenae* and evaluated its phylogenetic position within *Dianthus.* The plastome exhibited a typical circular quadripartite structure with a total length of 149,567 bp, comprising a large single-copy (LSC) region of 82,856 bp, a small single-copy (SSC) region of 17,105 bp, and a pair of inverted repeats (IRs) of 24,803 bp each. The genome contained the typical set of chloroplast genes, including protein-coding genes, transfer RNAs, and ribosomal RNAs, with duplicated genes located in the IR regions. Phylogenetic analysis based on complete chloroplast genome sequences strongly supported the placement of *D. helenae* within *Dianthus* and recovered it as a distinct lineage relative to other sampled species. Sliding window analysis of nucleotide diversity revealed uneven sequence variation across the plastome, with higher variability in the SSC and LSC regions than in the IRs. Several highly variable loci, including *trnK-UUU *, *rps16–trnQ-UUG *, *rpl32*, *ycf1*, and ndh-associated regions, were identified as potential molecular markers. These results provide an important genomic resource for *Dianthus* and establish a foundation for future phylogenetic, taxonomic, conservation, and molecular identification studies of this endemic Central Asian species.

## Introduction

*Dianthus* L. is one of the major genera of Caryophyllaceae and represents a taxonomically complex and evolutionarily important lineage within Caryophyllales. Recent taxonomic work has provided the first broad phylogenetic framework for the genus using plastid markers together with nuclear ITS data and has recognized 384 accepted species, confirming that *Dianthus* is far more diverse than traditionally assumed ( Fassou et al., 2022). At the same time, phylogenetic reconstruction in the genus remains challenging. Previous studies have shown that genetic distances among many *Dianthus* taxa are very low, species-level relationships are often weakly resolved, and plastid haplotypes may be deeply shared among related species, suggesting rapid radiation and a complex evolutionary history ( Fassou et al., 2022). More recent molecular analyses have nevertheless begun to recover major lineages within the genus. Combined plastid and nuclear datasets support the monophyly of *Dianthus* and indicate clear geographic and sectional structuring, while plastome-based analyses have resolved two strongly supported major clades among sampled species, demonstrating that chloroplast data are informative for understanding evolutionary relationships in the genus ( Lin et al., 2022; Mnxati & Mankga, 2025).

Beyond its phylogenetic importance, *Dianthus* is also notable for its ornamental, economic, and medicinal value. Several species are widely cultivated, while others are used in traditional medicine because they contain naturally occurring compounds with potential biological and medicinal activities ( Lin et al., 2022; Raman & Park, 2015; Meng et al., 2023). *Dianthus helenae* contains bioactive phytochemical compounds that may contribute to its medicinal potential ( Yusupova et al., 2022). The complete chloroplast genome provides a valuable reference for accurate species identification, authentication of plant materials, and the development of molecular markers for conservation studies.

In this context, complete chloroplast genomes provide a particularly useful source of evidence because they are generally conserved in structure and gene content, maternally inherited in most angiosperms, and widely used in comparative genomics, DNA marker development, and phylogenetic reconstruction ( Nikitina et al., 2025; Alieva et al., 2025; Tojiboeva et al., 2025; Dekhkonov et al., 2025; Ergashov et al., 2026a, b, c). Previous plastome studies in *Dianthus* have shown that chloroplast genomes are structurally conserved and phylogenetically informative, but taxon sampling remains limited and is still insufficient to fully clarify relationships across the genus ( Lin et al., 2022; Yang et al., 2021). *Dianthus helenae* Vved., an accepted species originally described in 1941, remains poorly studied at the genomic level ( Fassou et al., 2022). Therefore, characterization of its complete chloroplast genome will not only provide a new genomic resource for the genus, but will also help clarify its phylogenetic placement within *Dianthus*, contribute to comparative plastome studies, and support future taxonomic and conservation research on this endemic medicinal species from the Nuratau Mountains of Uzbekistan.

## Methods

**Figure 1.**
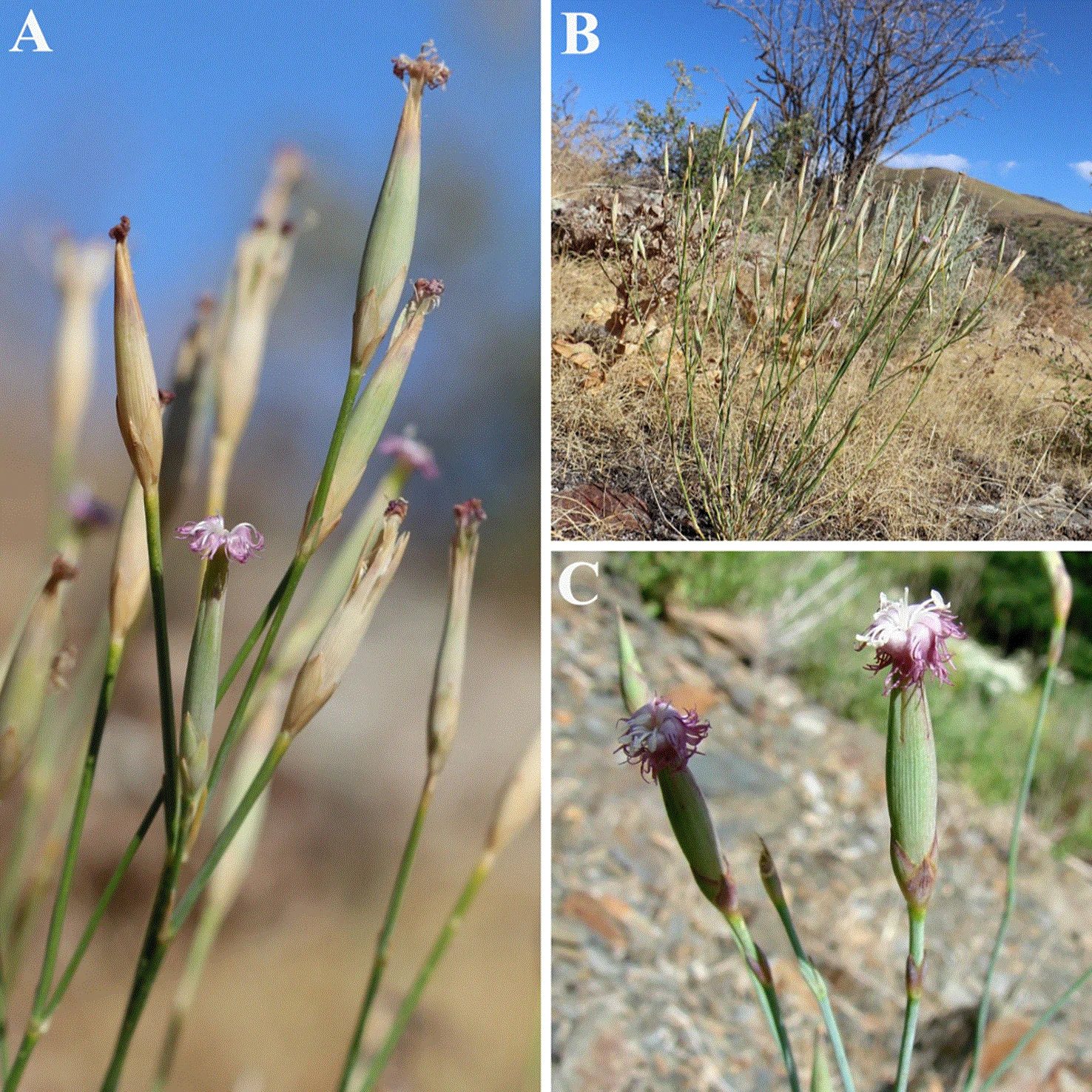
Habit of *Dianthus helenae* in the natural habitat. A – Close-up of reproductive shoots of *D. helenae*; B – Whole plant in its natural habitat; C – Close-up of flowering individuals. Various individuals of *D. helenae* in a shrub–grassland-steppe community on rocky and fine-soiled slopes in the Ustuksay area, Nuratau Range, Uzbekistan (approximately 1450 m a.s.l.; 4 July 2024; photograph by N. Beshko).

Fresh leaves of *Dianthus helenae* Vved. were collected from a wild population in the Nuratau Range, Uzbekistan ( Figure 1). Species identification was carried out by N. Beshko, and the voucher specimen is deposited in the National Herbarium of Uzbekistan (TASH) under accession number TASH139868.

Total genomic DNA was extracted from approximately 100 mg of fresh leaf tissue using a Tiangen Plant Genomic DNA Kit (Tiangen Biotech Co., Beijing, China; Cat. No. DP305) following the manufacturer’s protocol. Sequencing libraries were prepared using ~1 μg of purified DNA with the NEBNext Ultra II DNA Library Prep Kit for Illumina (New England Biolabs, USA; Cat. No. E7645S), including fragmentation to ~350 bp, end repair, adapter ligation, and PCR amplification. Library quality and fragment size distribution were assessed using an Agilent 5400 system. Qualified libraries were sequenced on an Illumina platform at Novogene Bioinformatics Technology Co.

Raw reads were first quality-filtered to remove adapter sequences, low-quality reads, and reads containing ambiguous bases. The resulting clean reads were then used for de novo plastome assembly with NOVOPlasty ( Dierckxsens et al., 2017). Gene annotation was performed in Geneious Prime using *Dianthus chinensis* (GenBank accession OP136025) as the reference genome and manually verified for start and stop codons as well as exon–intron boundaries ( Kearse et al., 2012). A circular chloroplast genome map was generated using OGDRAW ( Greiner et al., 2019). The assembled chloroplast genome was deposited in GenBank under accession number PZ251004.

Nucleotide diversity (Pi) was calculated using DnaSP v6.12.03 based on the aligned plastome sequences of eight *Dianthus* species, including *D. helenae* ( Rozas et al., 2017). A sliding-window analysis was performed with a window length of 1000 bp and a step size of 500 bp.

For phylogenetic reconstruction, 22 plastome sequences representing nine *Dianthus* species and one outgroup taxon ( *Petrorhagia saxifraga*) were retrieved from NCBI GenBank ( Table 1). Complete chloroplast genomes were aligned using MAFFT ( Katoh & Standley, 2013). Maximum likelihood analysis was performed in RAxML with 1,000 bootstrap replicates ( Stamatakis, 2014). The GTR + G model was selected using jModelTest v2.1.4 under the Akaike Information Criterion ( Darriba et al., 2012).

**Table 1.** *Dianthus* chloroplast genome sequences used in this study. Bold accession means sequenced sample for this study.

No.	Organism	Accession
1	*Dianthus nudiflorus*	OP_353926
2	*Dianthus nudiflorus*	NC_087621
3	***Dianthus helenae***	**PZ251004**
4	*Dianthus longicalyx*	MT_001881
5	*Dianthus longicalyx*	NC_050834
6	*Dianthus longicalyx*	KM_668208
7	*Dianthus chinensis*	OP_136025
8	*Dianthus chinensis*	OP_136018
9	*Dianthus chinensis*	OP_136016
10	*Dianthus superbus*	OP_136023
11	*Dianthus superbus*	NC_082201
12	*Dianthus barbatus*	NC_082202
13	*Dianthus barbatus*	OP_136024
14	*Dianthus barbatus*	OP_136026
15	*Dianthus cincinnatus*	OP_136020
16	*Dianthus gratianopolitanus*	LN_877389
17	*Dianthus gratianopolitanus*	LN_877395
18	*Dianthus gratianopolitanus*	LN_877393
19	*Dianthus caryophyllus*	MG_989277
20	*Dianthus caryophyllus*	NC_039650
21	*Dianthus caryophyllus*	OP_136027
22	*Dianthus caryophyllus*	KU_904222
23	*Petrorhagia saxifraga*	OP_353913

## Results

**Figure 2.**
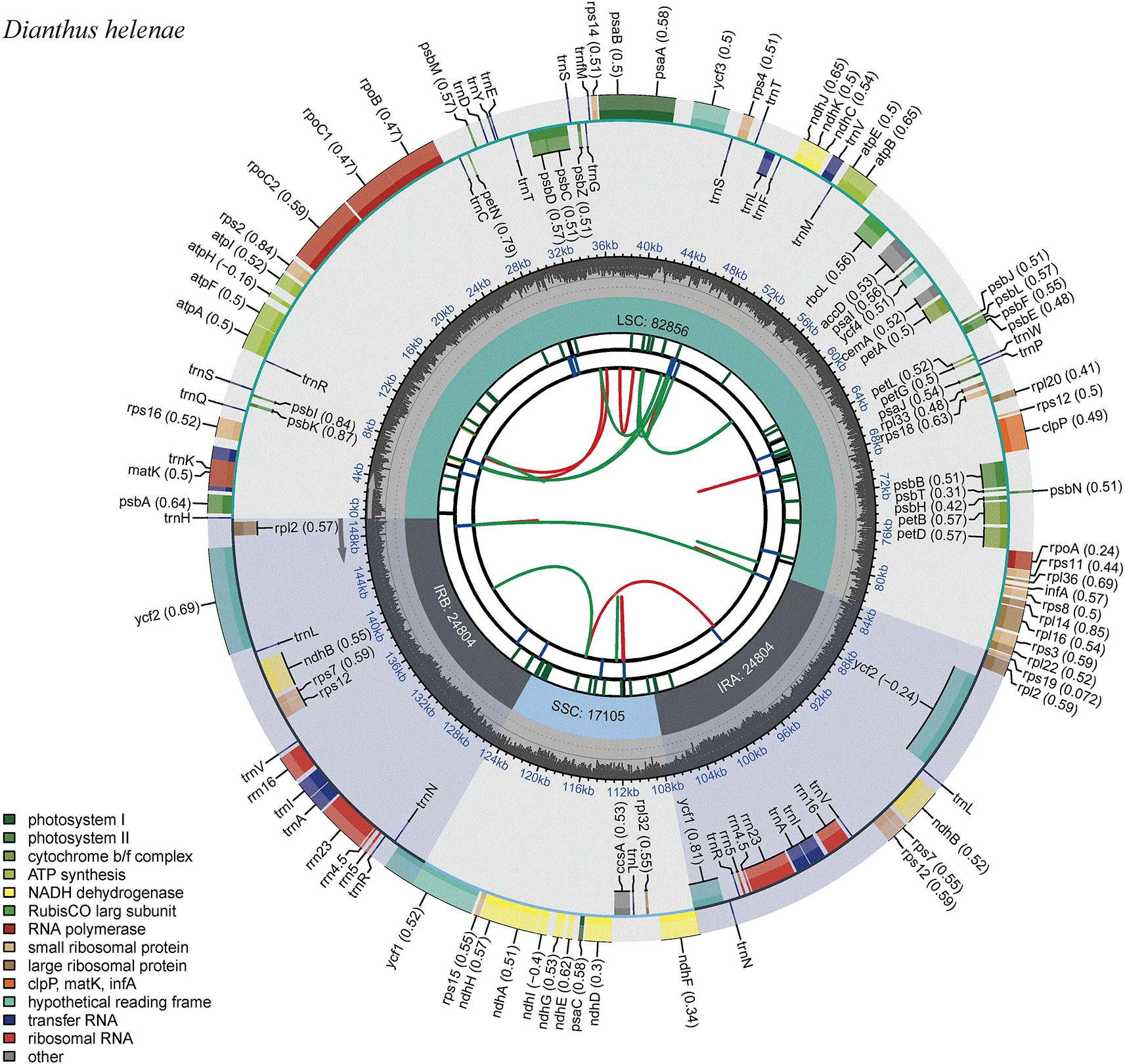
Circular map of the chloroplast genome of *Dianthus helenae.* From the center outward, the map consists of six concentric rings. The innermost ring shows forward and reverse repeats, connected by red and green arcs, respectively. The second ring indicates tandem repeats, while the third ring displays microsatellite sequences. The fourth ring shows the sizes of the large single-copy (LSC), small single-copy (SSC), and two inverted repeat regions (IRa and IRb). The fifth ring represents the distribution of GC content. The outermost ring displays genes grouped according to their functional categories with the corresponding codon usage bias values shown in parentheses after each gene name.

The chloroplast genome map of *Dianthus helenae* revealed a typical circular quadripartite structure, consisting of a large single-copy (LSC) region of 82,856 bp, a small single-copy (SSC) region of 17,105 bp, and a pair of inverted repeats (IRa and IRb) of 24,803 bp each, giving a total genome length of 149,567 bp ( Figure 2). The genome contained the usual set of plastid genes involved in photosynthesis, ATP synthesis, transcription, and translation, including protein-coding genes, transfer RNAs, and ribosomal RNAs. As shown in the map, genes were distributed on both strands, while several genes located in the IR regions were duplicated, including rRNA and some tRNA/protein-coding genes. This agrees well with comparative studies showing that *Dianthus* chloroplast genomes are highly uniform in overall architecture, generally falling within a narrow size range and containing largely conserved gene sets, with only limited variation at IR/SC boundaries ( Lin et al., 2022; Meng et al., 2023; Wicke et al., 2011; Daniell et al., 2016; Yang et al., 2021).

**Figure 3.**
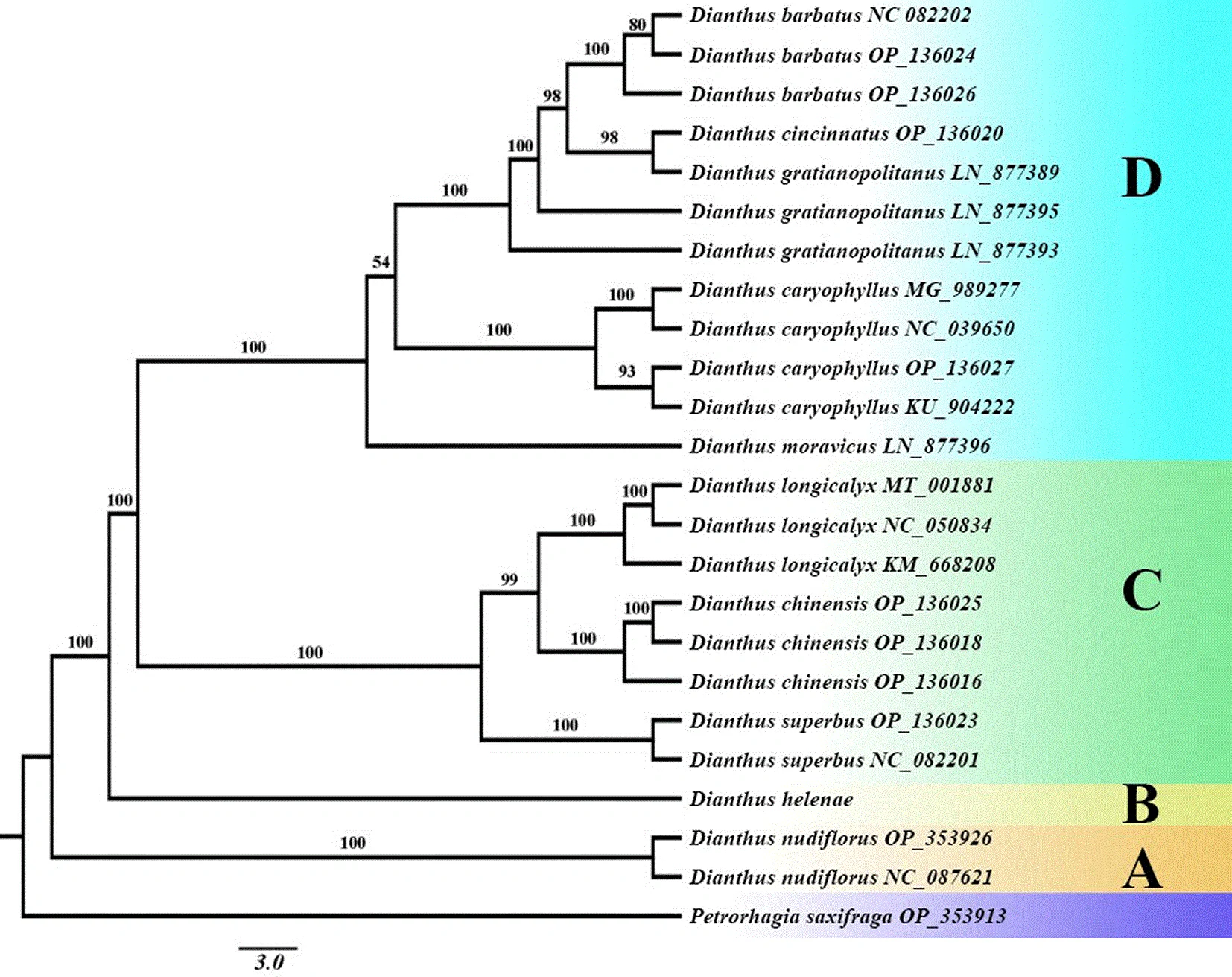
Maximum-likelihood tree of *Dianthus* plastomes. Maximum likelihood phylogenetic tree of *Dianthus* species based on complete chloroplast genome sequences. Numbers at nodes indicate bootstrap support values. Different colors (A–D) indicate the four major clades identified in the phylogenetic tree. Petrohragia saxifraga was used as the outgroup. The scale bar represents substitutions per site.

Phylogenetic analysis based on complete chloroplast genome sequences strongly supports the placement of *D. helenae* within the genus *Dianthus* ( Figure 3). The maximum likelihood tree revealed four major clades (A–D) within the genus *Dianthus*. *Dianthus helenae* formed a separate and well-supported lineage (Clade B), while the remaining species were grouped into three additional clades (A, C, and D). All major clades received strong bootstrap support (≥100), indicating robust phylogenetic relationships among the analyzed taxa.

The clustering pattern is consistent with previous plastome-based phylogenies, confirming the reliability of whole chloroplast genome data fogeir resolving relationships within Caryophyllaceae. The distinct positioning of *D. helenae* suggests its independent evolutionary trajectory within the genus. This is broadly compatible with previous whole-plastome analyses, which also resolved two major, well-supported chloroplast groups within *Dianthus*, one centered on *D. caryophyllus*, *D. barbatus*, and *D. gratianopolitanus*, and the other on *D. superbus*, *D. chinensis*, and *D. longicalyx* ( Lin et al., 2022).

**Figure 4.**
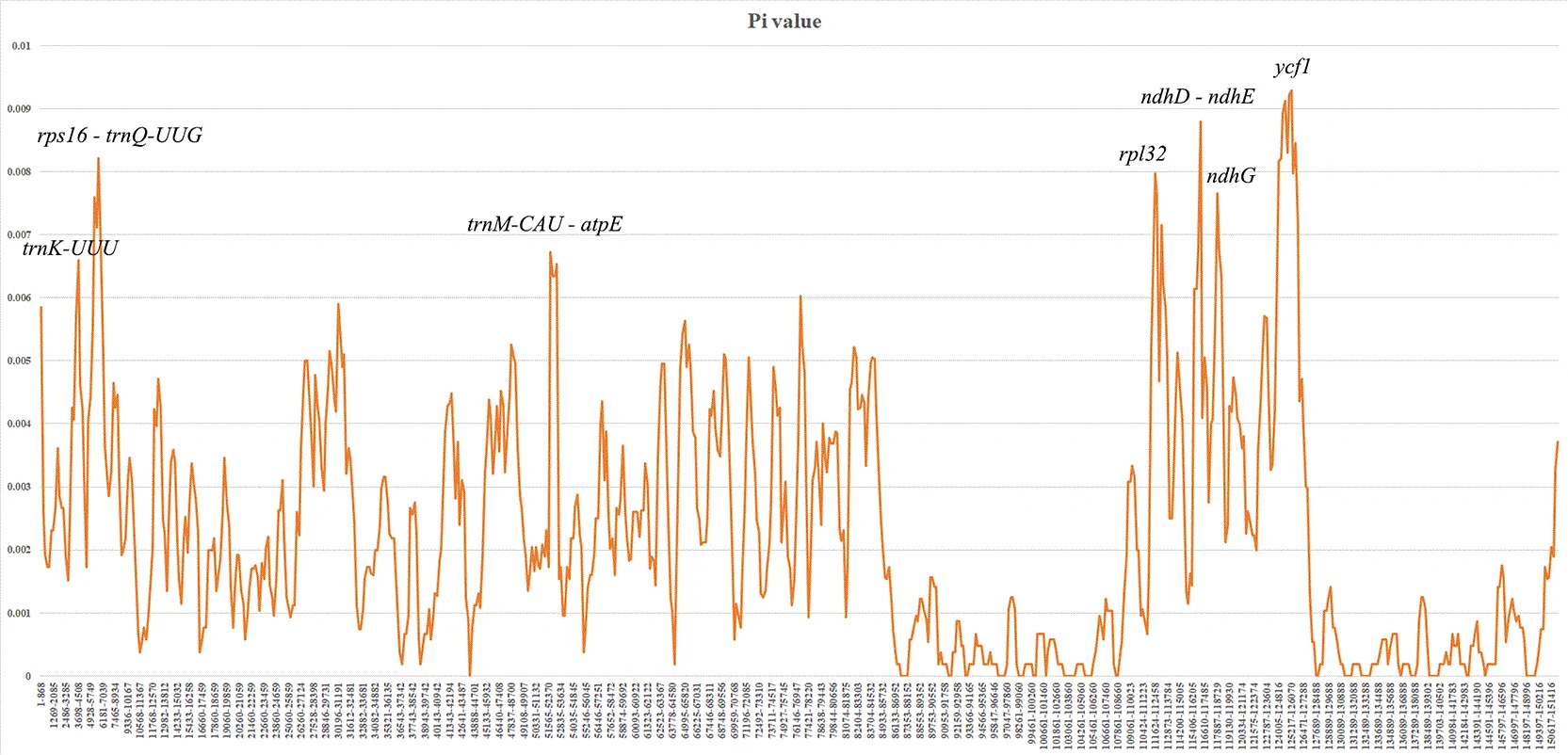
Sliding window analysis of nucleotide diversity across *Dianthus* chloroplast genomes. Nucleotide variability (Pi) is plotted along the genome sequence. The window length was set to 800 bp with a step size of 200 bp.

Sliding window analysis of nucleotide diversity (Pi) revealed heterogeneous variation across the plastome ( Figure 4). The SSC region exhibited higher variability compared to LSC and IR regions, consistent with patterns observed in other angiosperms. Several highly variable regions were identified, including coding regions such as *ycf1, rpl32* and intergenic spacers *ndhD-ndhE, rps16-trnQ-UUG.* These hotspots represent potential candidate regions for molecular marker development and phylogeographic studies. In contrast, IR regions showed significantly lower nucleotide diversity, reflecting their conserved nature and the stabilizing effect of gene duplication.

Overall, the present results show that *D. helenae* combines strong plastome structural conservatism with enough sequence divergence in a small number of highly informative loci to support phylogenetic placement and future species-level identification. Because *D. helenae* is an accepted Central Asian species of medicinal interest and several phytoecdysteroids have already been reported from it, the plastome resource generated here should be valuable for molecular authentication, population-level marker development, and future conservation-oriented studies of the genus in the region ( Yusupova et al., 2022).

## Ethics and consent

This study did not involve human participants or animals. Ethical approval and informed consent were therefore not required.

## Data Availability

NCBI GenBank: *Dianthus helenae* chloroplast genome. Accession number PZ251004. The accession numbers of comparative chloroplast genomes used in this study are provided in Table 1. No custom code was used in this study. Software used in the analysis included NOVOPlasty, Geneious Prime, OGDRAW, DnaSP v6.12.03, MAFFT, RAxML, and jModelTest v2.1.4, as cited in the Methods section.
