# Supplementary material for: The complete Chloroplast Genome of Dianthus Helenae, an Endemic Species with Medicinal Potential from the Nuratau Mountains, Uzbekistan
Source: F1000Res. 2026 Aug 21;15:727. Originally published 2026 May 13. [Version 4] doi: 10.12688/f1000research.180038.4 (PMC13562929; doi:10.12688/f1000research.180038.4)
Supplement: Peer review history

# Reviewer report: Reviewer response for version 4

**DOI:** 10.5256/f1000research.208238.r513354

**Published:** 2026-08-31

**Authors:** Hoang Dang Khoa Do

**Affiliations:** 1 Nguyen Tat Thanh University, Ho Chi Minh City, Ho Chi Minh, Vietnam

Dear Authors,

 Thank you very much for revising the manuscript based on the reviewer's comments.

 I do not have more comments about the revised manuscript.

 Best regards.

Are the datasets clearly presented in a usable and accessible format, and the assembly and annotation available in an appropriate subject-specific repository?

Yes

Are sufficient details of the sequencing and extraction, software used, and materials provided to allow replication by others?

Yes

Are the rationale for sequencing the genome and the species significance clearly described?

Yes

Are the protocols appropriate and is the work technically sound?

Yes

Reviewer Expertise:

Chloroplast genome evolution.

I confirm that I have read this submission and believe that I have an appropriate level of expertise to confirm that it is of an acceptable scientific standard.

# Reviewer report: Reviewer response for version 3

**DOI:** 10.5256/f1000research.206857.r506146

**Published:** 2026-07-31

**Authors:** Hoang Dang Khoa Do

**Affiliations:** 1 Nguyen Tat Thanh University, Ho Chi Minh City, Ho Chi Minh, Vietnam

Dear Authors,

 Thank you very much for revising the manuscript based on the reviewer's comments.

 However, there are still some minor issues. The size of *Dianthus helenae* chloroplast genome in the manuscript is 149,569 bp but the size of PZ251004 on GenBank is 149,567 bp. Please check and correct the information.

 Also, please correct "intergenic spacers ndh-ndhE" in the Results.

 Please add the meaning of A,B,C,D to Figure 3 legend because they were not mentioned in the main text...

 Best regards.

Are the datasets clearly presented in a usable and accessible format, and the assembly and annotation available in an appropriate subject-specific repository?

Yes

Are sufficient details of the sequencing and extraction, software used, and materials provided to allow replication by others?

Yes

Are the rationale for sequencing the genome and the species significance clearly described?

Yes

Are the protocols appropriate and is the work technically sound?

Yes

Reviewer Expertise:

Chloroplast genome evolution.

I confirm that I have read this submission and believe that I have an appropriate level of expertise to confirm that it is of an acceptable scientific standard, however I have significant reservations, as outlined above.

# Response

**Published:** 2026-08-07

**Authors:** Ibrokhimjon Ergashov

**Affiliations:** Botany, Institute of Botany of the Academy of Sciences of the Republic of Uzbekistan, Tashkent, Tashkent Province, Uzbekistan

Thank you very much for your careful review and valuable comments. We sincerely appreciate your suggestions, which have helped us further improve the quality and accuracy of our manuscript. We have carefully addressed all the minor issues raised and made the necessary corrections as follows:

**Comment 1:** *The size of Dianthus helenae chloroplast genome in the manuscript is 149,569 bp but the size of PZ251004 on GenBank is 149,567 bp. Please check and correct the information.*

**Response:** Thank you for pointing out this discrepancy. We rechecked the chloroplast genome information and corrected the genome size of *Dianthus helenae* in the manuscript to 149,567 bp, consistent with the GenBank accession PZ251004.

**Comment 2:** *Please correct "intergenic spacers ndh-ndhE" in the Results.*

**Response:** Thank you for your suggestion. We have corrected this terminology throughout the manuscript. The phrase " *ndh-ndhE*" has been revised to " *ndhD–ndhE* " in the Results section.

**Comment 3:** *Please add the meaning of A, B, C, D to Figure 3 legend because they were not mentioned in the main text.*

**Response:** Thank you for this helpful suggestion. We have revised the legend of Figure 3 by adding an explanation of the four major clades (A–D) indicated in the phylogenetic tree. The revised figure legend now clarifies that A–D represent the four major clades identified in the phylogenetic analysis.

 We sincerely appreciate the reviewer’s constructive comments and believe that these revisions have improved the clarity and accuracy of our manuscript.

# Reviewer report: Reviewer response for version 2

**DOI:** 10.5256/f1000research.202331.r491239

**Published:** 2026-06-19

**Authors:** Ruslan Kalendar

**Affiliations:** 1 University of Helsinki, Helsinki, Finland

This genome note describes the sequencing, assembly, and characterization of the complete chloroplast genome of Dianthus helenae Vved., a species endemic to the Nuratau Mountains in Uzbekistan. This primarily descriptive study presents a single plastome sequence alongside basic comparative and phylogenetic analyses.

 Strengths: This study fills a significant gap in genomic resources for Central Asian Dianthus species. The assembled genome has been deposited in GenBank under accession number PZ251004, making the data publicly accessible. The methods are standard and well-described. The phylogenetic placement of D. helenae is consistent with previous whole-plastome analyses of the genus. Additionally, the sliding window analysis identified several candidate hypervariable loci that could be useful for developing future molecular markers.

 Concerns:

 There is a numerical inconsistency between the abstract and the results section regarding the total genome length (149,567 bp vs. 149,709 bp), the LSC size (82,856 bp vs. 82,836 bp), and the IR length (24,804 bp vs. 24,884 bp). This must be resolved before final approval.

 The phylogenetic dataset is limited to nine Dianthus species. Including a broader range of taxa would strengthen inferences about the position of D. helenae within the genus.

 The species's claimed medicinal relevance is mentioned in the title and discussion, yet it is only minimally addressed in the text. Elaborating briefly on the species' known phytochemical properties and explaining how the plastome resource could contribute to authentication or conservation efforts would improve the biological context.

 As noted by the previous reviewer, the numbers appearing after gene names in Figure 2 remain unexplained in the figure legend.

 Conclusion: As a genome note, the scope of the study is appropriate for F1000Research. However, the numerical inconsistencies between the abstract and results must be corrected, and minor clarifications to the figures and biological framing are needed before the article can be approved.

Are the datasets clearly presented in a usable and accessible format, and the assembly and annotation available in an appropriate subject-specific repository?

Yes

Are sufficient details of the sequencing and extraction, software used, and materials provided to allow replication by others?

Yes

Are the rationale for sequencing the genome and the species significance clearly described?

Yes

Are the protocols appropriate and is the work technically sound?

Yes

Reviewer Expertise:

molecular genetics, diversity and genomics

I confirm that I have read this submission and believe that I have an appropriate level of expertise to confirm that it is of an acceptable scientific standard.

# Response

**Published:** 2026-07-28

**Authors:** Ibrokhimjon Ergashov

**Affiliations:** Botany, Institute of Botany of the Academy of Sciences of the Republic of Uzbekistan, Tashkent, Tashkent Province, Uzbekistan

We sincerely thank the reviewers for the careful evaluation of our manuscript and for the constructive comments, which have helped improve the clarity and quality of the study. Below we provide point-by-point responses.

 Comment: 1/ The genome size and lengths of regions in the abstract and the results are different. Please check and revise.

**Response:** Thank you for pointing out this inconsistency. We carefully rechecked the genome size and the lengths of the genomic regions reported in the Abstract and Results sections. The incorrect values have been corrected, and the data are now consistent throughout the manuscript.

 Comment:2/ Please add the meaning of numbers after the gene names in Figure 2. Thank you.

**Response:** Thank you for your comment. The numbers in parentheses following each gene name indicate the corresponding codon usage bias values. We have added this explanation to the legend of Figure 2.

# Reviewer report: Reviewer response for version 2

**DOI:** 10.5256/f1000research.202331.r489969

**Published:** 2026-06-05

**Authors:** Hoang Dang Khoa Do

**Affiliations:** 1 Nguyen Tat Thanh University, Ho Chi Minh City, Ho Chi Minh, Vietnam

Dear Authors,

 Thank you very much for revising the manuscript based on the reviewer's comments.

 Please check my comments below.

 1/ The genome size and lengths of regions in the abstract and the results are different. Please check and revise.

 2/ Please add the meaning of numbers after the gene names in Figure 2.

 Thank you.

Are the datasets clearly presented in a usable and accessible format, and the assembly and annotation available in an appropriate subject-specific repository?

Yes

Are sufficient details of the sequencing and extraction, software used, and materials provided to allow replication by others?

Yes

Are the rationale for sequencing the genome and the species significance clearly described?

Yes

Are the protocols appropriate and is the work technically sound?

Yes

Reviewer Expertise:

Chloroplast genome evolution.

I confirm that I have read this submission and believe that I have an appropriate level of expertise to confirm that it is of an acceptable scientific standard, however I have significant reservations, as outlined above.

# Response

**Published:** 2026-07-28

**Authors:** Ibrokhimjon Ergashov

**Affiliations:** Botany, Institute of Botany of the Academy of Sciences of the Republic of Uzbekistan, Tashkent, Tashkent Province, Uzbekistan

We sincerely thank the reviewers for the careful evaluation of our manuscript and for the constructive comments, which have helped improve the clarity and quality of the study. Below we provide point-by-point responses.

 Comment: 1. There is a numerical inconsistency between the abstract and the results section regarding the total genome length (149,567 bp vs. 149,709 bp), the LSC size (82,856 bp vs. 82,836 bp), and the IR length (24,804 bp vs. 24,884 bp). This must be resolved before final approval.

**Response:** Thank you for pointing out this inconsistency. We carefully rechecked the genome size and the lengths of the genomic regions reported in the Abstract and Results sections. The incorrect values have been corrected, and the data are now consistent throughout the manuscript.

 Comment: 2. The phylogenetic dataset is limited to nine Dianthus species. Including a broader range of taxa would strengthen inferences about the position of D. helenae within the genus.

**Response:** Thank you for this valuable suggestion. We agree that including a broader taxonomic sampling would strengthen the phylogenetic inference. However, at the time of our analysis, only nine *Dianthus* species with suitable chloroplast genome sequences were available in public databases, and all of these sequences were included in our dataset.

 Comment: 3. The species's claimed medicinal relevance is mentioned in the title and discussion, yet it is only minimally addressed in the text. Elaborating briefly on the species' known phytochemical properties and explaining how the plastome resource could contribute to authentication or conservation efforts would improve the biological context.

**Response:** Thank you for this valuable comment. We have expanded the Introduction to provide additional biological context regarding the medicinal potential of *Dianthus helenae* .

 Comment: 4. As noted by the previous reviewer, the numbers appearing after gene names in Figure 2 remain unexplained in the figure legend.

**Response:** Thank you for your comment. The numbers in parentheses following each gene name indicate the corresponding codon usage bias values. We have added this explanation to the legend of Figure 2.

# Reviewer report: Reviewer response for version 1

**DOI:** 10.5256/f1000research.198612.r485873

**Published:** 2026-05-21

**Authors:** Hoang Dang Khoa Do

**Affiliations:** 1 Nguyen Tat Thanh University, Ho Chi Minh City, Ho Chi Minh, Vietnam

Dear Authors,

 Thank you very much for adding new genomic data about Dianthus helenae.

 The content of the manuscript is suitable for Genome Note of F1000Research.

 Please check my comments below.

 1/"Dianthus helenae Vved., an accepted species originally described in 1941..." In this sentence, please use italic font for helenae.

 2/ "Clean reads were assembled using NOVOPlasty" Please add method for getting clean reads from raw reads.

 3/ "Nucleotide diversity (Pi) was calculated using DnaSP v6.12.03" Please add information about the number of Dianthus species for nucleotide diversity analysis.

 4/ Please add A, B, C for three parts of Figure 1 and their subtitles.

 5/ The left side of Figure might be accidentally cut. Please use the original figure. Also, please add more details about the figure (e.g., meaning of other circles, the inner read, green lines, numbers after gene names, etc...).

 Thank you.

Are the datasets clearly presented in a usable and accessible format, and the assembly and annotation available in an appropriate subject-specific repository?

Yes

Are sufficient details of the sequencing and extraction, software used, and materials provided to allow replication by others?

Yes

Are the rationale for sequencing the genome and the species significance clearly described?

Yes

Are the protocols appropriate and is the work technically sound?

Yes

Reviewer Expertise:

Chloroplast genome evolution.

I confirm that I have read this submission and believe that I have an appropriate level of expertise to confirm that it is of an acceptable scientific standard, however I have significant reservations, as outlined above.

# Response

**Published:** 2026-05-22

**Authors:** Ibrokhimjon Ergashov

**Affiliations:** Botany, Institute of Botany of the Academy of Sciences of the Republic of Uzbekistan, Tashkent, Tashkent Province, Uzbekistan

We sincerely thank the reviewer for the careful evaluation of our manuscript and for the

 constructive comments, which have helped improve the clarity and quality of the study.

 Below we provide point-by-point responses. 

**Comment:** 1/"Dianthus helenae Vved., an accepted species originally described in 1941..." In this sentence, please use italic font for helenae.

**Response**: Thank you for pointing this out. We have corrected the formatting and italicized the specific epithet in *Dianthus helenae* throughout the manuscript.

**Comment: **2/ "Clean reads were assembled using NOVOPlasty" Please add method for getting clean reads from raw reads.

**Response**: Thank you for this suggestion. We have added a description of the read-cleaning procedure before the assembly step. The revised sentence now reads:

 “Raw reads were first quality-filtered to remove adapter sequences, low-quality reads, and reads containing ambiguous bases. The resulting clean reads were then used for de novo plastome assembly with NOVOPlasty.”

**Comment: **3/ "Nucleotide diversity (Pi) was calculated using DnaSP v6.12.03" Please add information about the number of Dianthus species for nucleotide diversity analysis.

**Response**: Thank you for the suggestion. We have added the number of *Dianthus* species included in the nucleotide diversity analysis. The revised sentence now reads:

 “Nucleotide diversity (Pi) was calculated using DnaSP v6.12.03 based on the aligned plastome sequences of eight *Dianthus* species, including *D. helenae*.”

**Comment: **4/ Please add A, B, C for three parts of Figure 1 and their subtitles.

**Response**: Thank you for this valuable suggestion. We have revised Figure 1 by adding panel labels **A–C** to the three image parts and updated the caption accordingly. The revised caption now reads:

**Figure 1. Habit of** *Dianthus helenae* **in its natural habitat.**

**A** – Close-up of reproductive shoots of *D. helenae*;

**B** – Whole plant in its natural habitat;

**C** – Close-up of flowering individuals.

 Various individuals of *D. helenae* in a shrub–grassland-steppe community on rocky and fine-soiled slopes in the Ustuksay area, Nuratau Range, Uzbekistan (approximately 1450 m a.s.l.; 4 July 2024; photograph by N. Beshko).

**Comment: **5/ The left side of Figure might be accidentally cut. Please use the original figure. Also, please add more details about the figure (e.g., meaning of other circles, the inner read, green lines, numbers after gene names, etc...).

**Response:** Thank you for this helpful comment. We have replaced the cropped figure with the original version and revised the caption to provide more detailed explanations of the plastome map, including the LSC, SSC, IRa and IRb regions, transcription directions, color-coded gene functional groups, GC/AT content, region boundaries, GC/AT skew curves, and the numbers shown after some gene names.
